# The Romanian context: specificity and challenges of human resources in tourism

**Published:** 2014

**Authors:** Albu Carmen Cristina

**Affiliations:** * Transilvania University Braşov, Brașov, Romania

**Abstract**

Tourism represents a service-oriented activity of limited capitalist intensity, therefore the human resources play an essential role both in the competitiveness of the organizations and in the attractiveness of the destinations as far as, among others, the quality of reception and the performance are concerned. The tourism goods primarily represent a pleasant experience and consequently the human factor becomes a crucial component for the manner the consumer perceives the service quality.

The major challenge for the field of tourism resides in the qualification and the availability of the seasonal workplaces and their ability to meet the quality objectives required by the visitors. Therefore, innovation in terms of organization, formation, and governance represents a crucial factor for the competitiveness development of the actors involved in this field. The priority is determined by the acquiring of the needed skills, the management of these skills, and by the continuous formation allowing the human resources to become a competitive gain in terms of performance and quality. Even more than in other fields, the knowledge management represents a primordial axis of organizational competitiveness and innovation, a catalyst for the development of knowledge sharing and management, as this management encourages professional openness, synergy identification and promotion, easier access to knowledge, competence and expertise.

This work proposes an analysis of prominent scientific studies published in USA, Canada, and the European Union and developed by international organisms involved in the tourism development, in order to highlight the dominant influences of sustainable development, social responsibility and quality principles within this representative field of socio-economic activity. The present work also highlights the workplace and its field oriented skill characteristics, factors and the tendencies influencing the competence shortage, the policies involved in attenuating the human resources deficiency in global tourism. The transition to Romanian tourism proposes the highlighting of structural features and differences related to countries with a tradition in this field and a diagnose analysis able to represent a point of entry for the recovery and rectification of disparities.

**Keywords:** human resources, tourism, organization

